# Exploring the multifaceted therapeutic mechanism of Schisanlactone E (XTS) in APP/PS1 mouse model of Alzheimer’s disease through multi-omics analysis

**DOI:** 10.3389/fmicb.2024.1440564

**Published:** 2024-07-09

**Authors:** Zhenyan Song, Jiawei He, Wenjing Yu, Chunxiang He, Miao Yang, Ping Li, Ze Li, Gonghui Jian, Shaowu Cheng

**Affiliations:** ^1^School of Integrated Chinese and Western Medicine, Hunan University of Chinese Medicine, Changsha, Hunan, China; ^2^Key Laboratory of Hunan Province for Integrated Traditional Chinese and Western Medicine on Prevention and Treatment of Cardio-Cerebral Diseases, College of Integrated Traditional Chinese and Western Medicine, Hunan University of Chinese Medicine, Changsha, Hunan, China; ^3^Hunan University of Chinese Medicine, The First Hospital of Hunan University of Chinese Medicine, Changsha, Hunan, China

**Keywords:** Schisanlactone E, Alzheimer’s disease, 16S rDNA, metabolomics, Akkermansia, 4-methylcatechol, microbial-gut-brain axis

## Abstract

**Background:**

Schisanlactone E, also known as XueTongSu (XTS), is an active compound extracted from the traditional Tujia medicine Kadsura heteroclita (“XueTong”). Recent studies highlight its anti-inflammatory and antioxidant properties, yet the mechanisms of XTS’s therapeutic effects on Alzheimer’s disease (AD) are unclear. This study aims to elucidate the therapeutic efficacy and mechanisms of XTS in AD.

**Methods:**

Ten C57BL/6 mice were assigned to the control group (NC), and twenty APP/PS1 transgenic mice were randomly divided into the model group (M) (10 mice) and the XTS treatment group (Tre) (10 mice). After an acclimatization period of 7 days, intraperitoneal injections were administered over a 60-day treatment period. The NC and M groups received saline, while the Tre group received XTS at 2 mg/kg. Learning and memory abilities were assessed using the Morris Water Maze (MWM) test. Histopathological changes were evaluated using hematoxylin and eosin (HE) and Nissl staining, and immunofluorescence was used to assess pathological products and glial cell activation. Cytokine levels (IL-1β, IL-6, TNF-α) in the hippocampus were quantified by qPCR. 16S rDNA sequencing analyzed gut microbiota metabolic alterations, and metabolomic analysis was performed on cortical samples. The KEGG database was used to analyze the regulatory mechanisms of XTS in AD treatment.

**Results:**

XTS significantly improved learning and spatial memory in APP/PS1 mice and ameliorated histopathological changes, reducing Aβ plaque aggregation and glial cell activation. XTS decreased the expression of inflammatory cytokines IL-1β, IL-6, and TNF-α. It also enhanced gut microbiota diversity, notably increasing Akkermansia species, and modulated levels of metabolites such as isosakuranetin, 5-KETE, 4-methylcatechol, and sphinganine. Pathway analysis indicated that XTS regulated carbohydrate metabolism, neuroactive ligand-receptor interactions, and alanine, aspartate, and glutamate metabolism, mitigating gut microbiota dysbiosis and metabolic disturbances.

**Conclusion:**

XTS ameliorates cognitive deficits, pathological changes, and inflammatory responses in APP/PS1 mice. It significantly modulates the gut microbiota, particularly increasing Akkermansia abundance, and influences levels of key metabolites in both the gut and brain. These findings suggest that XTS exerts anti-AD effects through the microbial-gut-brain axis (MGBA).

## 1 Introduction

Alzheimer’s Disease (AD) is a prevalent neurodegenerative disorder associated with aging, with its incidence rising steadily alongside global population aging (Breijyeh and Karaman, 2020). The pathogenesis of AD remains highly debated, with key pathological hallmarks including amyloid-beta (Aβ) plaque deposition, neurofibrillary tangles (NFTs) formed from hyperphosphorylated tau protein, neuronal loss, and chronic neuroinflammation (Scheltens et al., 2021). Thus, elucidating the mechanisms of AD onset, developing preventative measures, and formulating effective treatments are of paramount importance.

Schisanlactone E, also known as XueTongSu (XTS), is an active compound derived from the traditional Tujia medicine Kadsura heteroclita, commonly referred to as “XueTong” (Wang et al., 2021).”XueTong” has a long history of medicinal use among the Tujia people of China, with well-documented clinical efficacy. Recent research highlights the significant anti-inflammatory and antioxidant properties of XTS (Liu et al., 2020). XTS inhibits the NF-κB signaling pathway by inhibiting the expression of RANKL (Receptor Activator for Nuclear Factor κ B Ligand) and its receptor RANK, thereby reducing the expression of inflammatory cytokines and diminishing inflammatory responses (Wang et al., 2006). Additionally, XTS can decrease the activity of matrix metalloproteinase-9 (MMP-9) and inhibit the IL-23/IL-17 inflammatory axis, thus reducing immune cell activation and inflammation (Zheng et al., 2024).

16S rDNA sequencing is a high-throughput technique used to analyze the composition of bacterial communities in specific environmental samples. It reveals the diversity, abundance, and structure of microbial populations and explores the relationships between microorganisms and their environment or host (Woo et al., 2008). Clinical studies have shown significant differences in the gut microbiota composition of AD patients compared to healthy elderly individuals (Liu et al., 2019; Pan et al., 2021),suggesting that modulating gut microbiota dysbiosis could be a promising therapeutic strategy for AD (Zhu et al., 2020; Zhang et al., 2023). Some plant-derived small molecules, such as Schisandra chinensis polysaccharide, have demonstrated anti-AD effects by altering the gut microbiota composition and subsequent metabolic changes in AD model rats (Fu et al., 2023). Therefore, investigating the impact of XTS on gut microbiota may uncover new therapeutic pathways for XTS in AD treatment.

Metabolomics enables the detection and quantification of numerous metabolites in tissues or biological fluids, revealing multiple network changes under disease conditions (Huo et al., 2020; Fang et al., 2021). Non-targeted mass spectrometry (MS) allows comprehensive exploration of metabolites within biological systems, providing a robust method for identifying and validating candidate metabolomic biomarkers (Su et al., 2023). Metabolomics supports a comprehensive understanding of alterations in the AD brain (Reveglia et al., 2021),aiding early screening and diagnosis through existing biomarkers (Lista et al., 2023). Combining gut microbiota, gut metabolites, and brain tissue metabolomic changes for joint analysis can provide insights into anti-AD drug mechanisms and support evidence for the Microbial-Gut-Brain Axis (MGBA) (Chen et al., 2024).

This study employed APP/PS1 double transgenic mouse models to investigate the pathological mechanisms of AD and the regulatory network mediated by XTS in AD treatment. We evaluated learning and memory improvements in APP/PS1 mice treated with XTS using the Morris Water Maze (MWM) test. HE staining, Nissl staining, and immunofluorescence assays observed the effects of XTS on brain tissue morphology and pathological products. 16S rDNA sequencing analyzed changes in gut microbiota abundance, while non-targeted metabolomics based on liquid chromatography-mass spectrometry (LC–MS) determined the regulatory effects of XTS on the cortical metabolic profile. Integrating gut microbiota-metabolite correlation analysis and the KEGG database allowed a multi-omic, multi-dimensional study of the anti-AD mechanisms of XTS. This comprehensive approach aims to further understand the anti-AD effects of XTS.

## 2 Materials

### 2.1 Schisanlactone E

**Figure 1:**
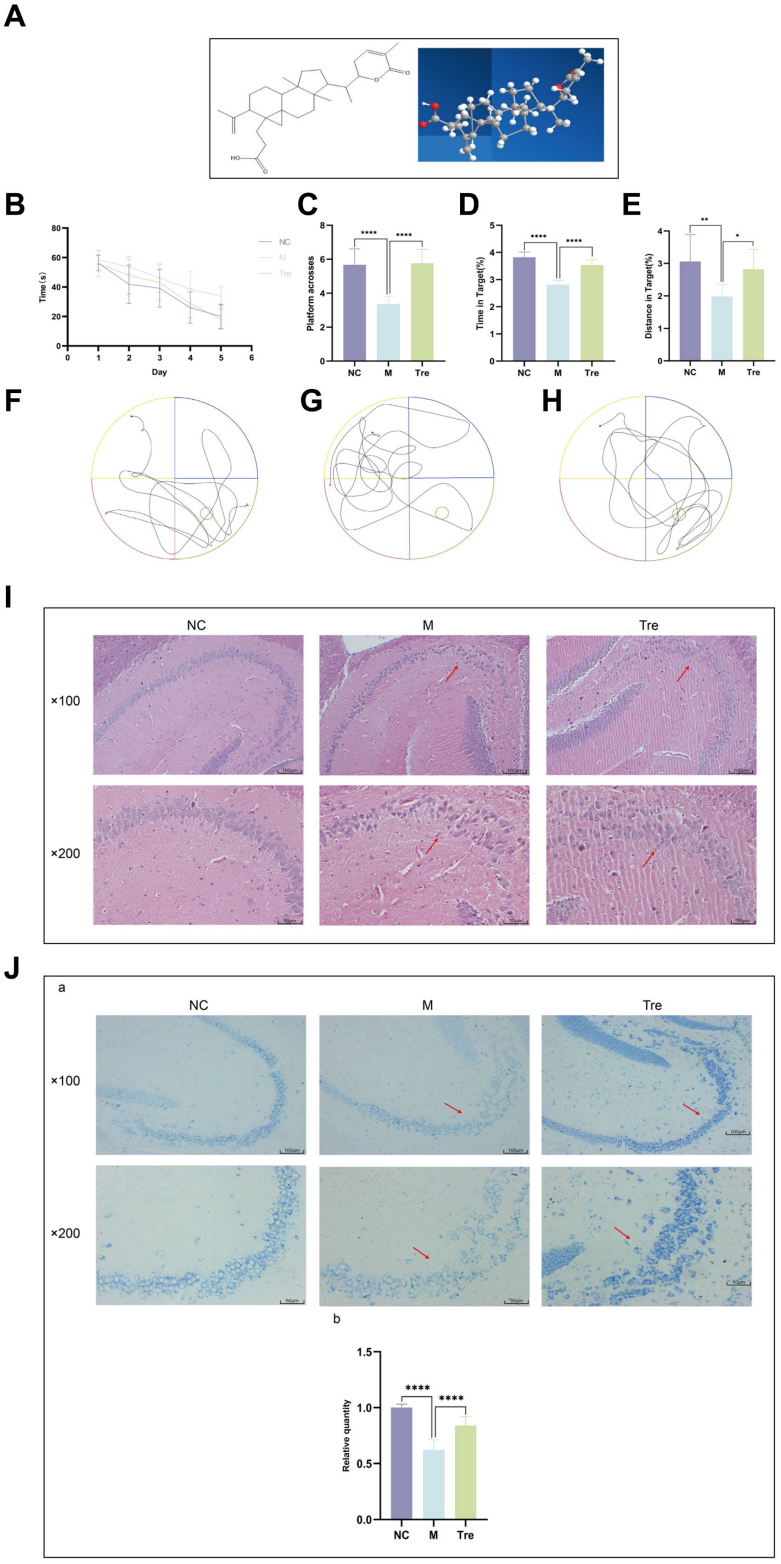
XTS improved the learning and memory functions and ameliorated the pathological changes in the brains of APP/PS1 mice. **(A)** XTS. **(B)** Latency period in the water maze. **(C)** Number of platform crossings. **(D)** Time spent in the platform area/total time. **(E)** Distance traveled in the platform area/total distance. **(F)** Trajectory plot of the NC group. **(G)** Trajectory plot of the M group. **(H)** Trajectory plot of the Tre group. **(I)** HE staining of mouse brain tissue. **(J)** ^a^Nissl staining of mouse brain tissue; ^b^the relative quantity of Nissl bodies. *p < 0.05; **p < 0.01; ***p < 0.001; ****p < 0.0001.

Schisanlactone E (also known as XTS, PubChem CID: 14844611, purity >99.99%; HPLC, Hunan, China) was prepared by the College of Pharmacy, Hunan University of Chinese Medicine. The molecular formula of Schisanlactone E is C_30_H_44_O_4_ (Figure 1A).

### 2.2 Animals

APP/PS1 double transgenic (APP/PS1) mice and C57BL/6 mice were purchased from Nanjing Junke Bioengineering Co., Ltd. The study involved 20 male APP/PS1 mice and 10 male C57BL/6 mice, each weighing between 25 and 35 grams. The animals were supplied with the experimental animal production license number SCXK(Su)2020–0009. They were housed in the Animal Experiment Center of Hunan University of Chinese Medicine. The experimental protocol was approved by the Ethics Committee of Hunan University of Chinese Medicine (Approval No. LLBH-2021090804). The 10 C57BL/6 mice were assigned to the control group (NC), and the 20 APP/PS1 mice were randomly divided into the model group (M) (10 mice) and the XTS-treated group (Tre) (10 mice). All animals had free access to water and were maintained at a constant temperature under a 12-h light/dark cycle with an adaptation period of 3 days.

### 2.3 Instruments and reagents

The following instruments and reagents were used in the study: SMART 3.0 small animal behavior recording and analysis system (RWD Life Science Co., Ltd.); Paraffin microtome (Thermo Fisher Scientific, USA); Al + confocal laser microscope (Nikon, Japan); TissueFAXS imaging system (Tissue Gnostics GmbH, Austria); High-speed refrigerated centrifuge (Eppendorf Centrifuge 5430 R); Centrifuge (Thermo Fisher Scientific, Heraeus Fresco17); Balance (Sartorius, BSA124S-CW); Refrigerated centrifuge (Hunan Xiangyi Experiment Equipment Co., H1850-R); Vortex mixer (Haimen Kylin-bell Lab Instruments Co., Ltd., BE-96); Tissue homogenizer (Zhejiang Meibi Experiment Equipment Co., Ltd., MB-96); Ultrasonic cleaner (Kunshan Shumei Experiment Equipment Co., Ltd., KW-100TDV); Filter membranes (Tianjin Jinteng Experiment Equipment Co., Ltd., 0.22 μm PTFE); Liquid chromatography system (Thermo, Vanquish); Mass spectrometer (Thermo, Orbitrap Exploris 120); Water (Millipore); Acetonitrile (Thermo, 75-05-8, purity 99.9%); Methanol (Thermo, 67-56-1, purity ≥99.0%); Chloroform (Sinopharm, 67-66-3, purity 99.9%); 2-chloro-L-phenylalanine (Aladdin, 103616-89-3, purity 98%); Formic acid (TCI, 64-18-6, LC-MS grade); Ammonium formate (Sigma, 540-69-2, purity ≥99.9%); 6E10 antibody (SIG-39320, Covance Inc., USA); Iba1 antibody (sc-32725, Santa Cruz Biotechnology, Inc.); PCR product purification kit (AMPure XT beads, Beckman Coulter Genomics, Danvers, MA, USA); PCR product quantification kit (Qubit, Invitrogen, USA).

## 3 Methods

### 3.1 Preparation of XTS

XTS was prepared by the College of Pharmacy at Hunan University of Chinese Medicine. The stems of Kadsura heteroclita were collected from Huping Mountain in Shimen County, Hunan Province, China. A voucher specimen (No. CEL 1280-KH) is preserved in the International Laboratory for Innovation and Development of Traditional Chinese Medicine and Ethnic Medicine at Hunan University of Chinese Medicine. Air-dried plant material (100 kg) was extracted with 80% ethanol under reflux to produce a viscous extract. XTS (PubChem CID: 14844611, purity >99.99%; HPLC, Hunan, China) was isolated and purified from the ethanol extract following the method described by Cao et al. (2020). HPLC analysis of the isolated XTS was performed using an Agilent 1,260 system equipped with a G1311C quaternary pump, G1329B sampler, G1316A column compartment, and G4212B diode array detector. An Agilent TC-C18 column (5 μm, 150 mm × 4.6 mm) was used with a mobile phase consisting of 0.1% phosphoric acid in water and acetonitrile. Gradient elution was carried out as follows: 0–30 min, 65–85% acetonitrile. The optimized flow rate, column temperature, injection volume, and detection wavelength were set at 1 mL/min, 25°C, 5 μL, and 210 nm, respectively (Cao et al., 2020; Yu et al., 2022; Zheng et al., 2024).

### 3.2 Animal grouping and drug intervention

Ten C57BL/6 mice were allocated to the NC group, and twenty APP/PS1 mice were randomly divided into two groups: the model group (M) and the treatment group (Tre). After a 7-day adaptation period, the mice received intraperitoneal injections for 60 days. The NC and M groups were administered an equivalent volume of physiological saline intraperitoneally. The XTS treatment group received intraperitoneal injections of XTS at a concentration of 2 mg/kg (Zheng et al., 2024).

### 3.3 Morris water maze test

The MWM test was employed to assess spatial learning and memory using the SMART 3.0 animal behavior recording and analysis system. The pool was divided into four quadrants, with the target quadrant being the first quadrant, where the escape platform was submerged 1 cm below the water surface. The MWM test consisted of place navigation and spatial probe trials. During place navigation, mice were released from each of the four quadrants in sequence, and the time taken to find and remain on the platform for 2 s was recorded. Each trial lasted a maximum of 60 s. If a mouse failed to find the platform within 60 s, it was guided to the platform and allowed to stay for 10 s to aid memory. Each mouse underwent one complete session per day, consisting of four trials, one from each quadrant. On the sixth day, the platform was removed for the spatial probe trial, and the time spent in the target quadrant and the number of platform crossings within 60 s were recorded.

### 3.4 Tissue collection

Mice were anesthetized with 3% pentobarbital sodium according to body weight, followed by fixation of the limbs and an abdominal U-shaped incision. Blood was collected from the eyeballs, approximately 1 mL per mouse. The heart was perfused with 20 mL of pre-cooled saline at 4°C via the left ventricle, with the right atrium cut open, until the viscera turned white, indicating successful perfusion. Brain tissues were collected, and the left and right hemispheres were separated. The left hemisphere was fixed in paraformaldehyde, while the right hemisphere was dissected into cortex and hippocampus, snap-frozen in liquid nitrogen, and stored at −80°C. At least five fecal pellets were collected from the rectum of each mouse, snap-frozen in liquid nitrogen, and stored at −80°C until further analysis. Blood samples were centrifuged at 3000 rpm for 15 min, and the serum was stored at −80°C.

### 3.5 Hematoxylin and eosin staining

The left hemisphere was fixed and embedded in paraffin. Sections were cut to a thickness of 3 μm using a paraffin microtome, deparaffinized, and rehydrated. Hematoxylin staining was performed for 5 min, followed by a water rinse. Differentiation was done using 0.5% hydrochloric acid in ethanol for 1 min, and bluing was achieved with PBS. Eosin staining was performed for 1 min, followed by dehydration in a graded alcohol series, air drying, and mounting with neutral gum.

### 3.6 Nissl staining

The left hemisphere was fixed, paraffin-embedded, and sectioned. After deparaffinization and rehydration, sections were stained with 1% toluidine blue at 37°C for 25 min. Differentiation was carried out in 95% ethanol for 30 s. Sections were then dehydrated in a graded alcohol series and mounted with neutral gum.

### 3.7 Immunofluorescence

Brain tissue samples were fixed in 4% paraformaldehyde and paraffin-embedded. Sections were cut to a thickness of 5 μm using a microtome (HM355S, Thermo Scientific, Shanghai, China). For immunostaining, sections were deparaffinized, subjected to antigen retrieval, blocked, and incubated with primary and secondary antibodies as previously described (Song et al., 2023). Antigen retrieval and tissue clearing were performed to enhance antigenicity. After blocking to reduce non-specific binding, sections were incubated with primary antibodies: 6E10 (SIG-39320, Covance Inc., USA, 1:200) for Aβ deposition, Iba1 (sc-32725, Santa Cruz Biotechnology, Inc., 1:50) for microglial expression, and GFAP (16825-1-AP, Proteintech, Inc., 1,200) for astrocytic expression. After primary antibody incubation, sections were incubated with appropriate secondary antibodies. Staining and mounting were performed according to established protocols. The TissueFAXS imaging system (Tissue Gnostics GmbH, Austria) was used for whole-slide imaging and subsequent statistical analysis, enabling comprehensive evaluation of stained tissue sections.

### 3.8 Quantitative real-time PCR (qPCR)

RNA was extracted from the left hippocampus using the TRIzo method, and RNA concentration was measured. The obtained RNA was stored at −80°C. For cDNA synthesis, the NovoScript® mRNA First-Strand cDNA Synthesis kit was used, with 1,000 ng RNA per 20 μL reaction volume. The synthesized cDNA was stored at −20°C. qPCR was conducted using SYBR Green to detect IL-1β, IL-6, and TNF-α mRNA expression levels, with β-actin as the internal control. Primer design was performed using Primer Premier 6.0, with Tm set at 55 ± 5°C and amplicon length between 100–300 bp (Table 1). Relative mRNA expression levels were calculated using the 2^-ΔΔCq^ method.

**Table 1:** Primer sequence.

Primer	Forward primer (5′ → 3′)	Reverse primer (3′ → 5′)	Length/bp
IL-1β	CTCATTGTGGCTGTGGAGAAG	ACACTAGCAGGTCGTCATCAT	148
IL-6	CCAGCCAGTTGCCTTCTTG	AATTAAGCCTCCGACTTGTGAA	139
TNF-α	AGATGTGGAACTGGCAGAGG	CACGAGCAGGAATGAGAAGAG	100
Actin	AGACCTCTATGCCAACACAGT	TCCTGCTTGCTGATCCACAT	210

### 3.9 16S rRNA gene sequencing analysis

#### 3.9.1 DNA extraction

Total genomic DNA was extracted from samples using the OMEGA Soil DNA Kit (M5635-02) (Omega Bio-Tek, Norcross, GA, USA) according to the manufacturer’s instructions and stored at −20°C until further analysis. The quantity and quality of the extracted DNA were assessed using a NanoDrop NC2000 spectrophotometer (Thermo Fisher Scientific, Waltham, MA, USA) and agarose gel electrophoresis, respectively.

#### 3.9.2 16S rRNA gene amplicon sequencing

The V3–V4 region of the bacterial 16S rRNA gene was amplified using the forward primer 338F (5’-ACTCCTACGGGAGGCAGCA-3′) and the reverse primer 806R (5’-GGACTACHVGGGTWTCTAAT-3′). Sample-specific 7-bp barcodes were incorporated into the primers for multiplex sequencing. The PCR reaction mixture contained 5 μL of 5× buffer, 0.25 μL of FastPfu DNA Polymerase (5 U/μL), 2 μL of dNTPs (2.5 mM), 1 μL of each forward and reverse primer (10 μM), 1 μL of DNA template, and 14.75 μL of ddH₂O. Thermal cycling conditions were: initial denaturation at 98°C for 5 min, followed by 25 cycles of denaturation at 98°C for 30 s, annealing at 53°C for 30 s, and extension at 72°C for 45 s, with a final extension at 72°C for 5 min. PCR amplicons were purified using Vazyme VAHTS DNA Clean Beads (Vazyme, Nanjing, China) and quantified with the Quant-iT PicoGreen dsDNA Assay Kit (Invitrogen, Carlsbad, CA, USA). Equal amounts of purified amplicons were pooled, and paired-end 2 × 250 bp sequencing was performed on the Illumina NovaSeq platform using the NovaSeq 6,000 SP Reagent Kit (500 cycles) at Suzhou PANOMIX Biomedical Tech Co., LTD.

#### 3.9.3 Sequence and statistical analysis

Microbiome bioinformatics analysis was performed using QIIME2 2019.4 (Bolyen et al., 2019) with modifications according to the official tutorials.^1^ Raw sequence data were demultiplexed using the demux plugin and primers were trimmed using the cutadapt plugin (Martin, 2011). Sequences were then quality-filtered, denoised, merged, and chimera-checked using the DADA2 plugin (Callahan et al., 2016). Non-singleton amplicon sequence variants (ASVs) were aligned with MAFFT (Katoh et al., 2002) and used to construct a phylogeny with FastTree2 (Price et al., 2009). Further analyses were conducted using QIIME2 and R packages (v3.2.0). Alpha diversity indices (Chao1, Observed species, Shannon, Simpson, Faith’s PD, Pielou’s evenness, and Good’s coverage) were calculated from the ASV table in QIIME2 and visualized as box plots. Beta diversity analyses, including Jaccard, Bray-Curtis, and UniFrac metrics, were performed to investigate microbial community variations and visualized via PCoA, NMDS, and UPGMA hierarchical clustering. Genus-level compositional profiles were analyzed using PCA. Taxonomy compositions and abundances were visualized using MEGAN (Huson et al., 2011) and GraPhlAn (Asnicar et al., 2015). Differentially abundant taxa were identified using LEfSe (Linear discriminant analysis effect size) and visualized as Manhattan plots (Segata et al., 2011). OPLS-DA (Orthogonal Partial Least Squares Discriminant Analysis) was used to reveal microbiota variations among groups, implemented with the R package “muma.” Random forest analysis was applied for sample classification using QIIME2. Microbial functions were predicted with PICRUSt2 (Douglas et al., 2020) using the KEGG database.

### 3.10 LC-MS analysis

#### 3.10.1 Sample preparation for LC-MS

Accurately weigh an appropriate amount of sample into a 2 mL centrifuge tube. Add 1,000 μL of extraction solution (75% methanol (9:1), 25% H₂O) and three steel balls. Homogenize the sample at 50 Hz for 60 s twice. Sonicate at room temperature for 30 min, followed by an ice bath for 30 min. Centrifuge at 12,000 rpm and 4°C for 10 min. Transfer the supernatant to a new 2 mL centrifuge tube, concentrate, and dry it. Redissolve the sample in 200 μL of 50% acetonitrile solution containing 2-Amino-3-(2-chlorophenyl)-propionic acid (4 ppm), filter through a 0.22 μm membrane, and transfer to LC–MS vials for analysis.

#### 3.10.2 LC-MS instrumental analysis

LC analysis was performed on a Vanquish UHPLC System (Thermo Fisher Scientific, USA) with an ACQUITY UPLC® HSS T3 column (150 × 2.1 mm, 1.8 μm) (Waters, Milford, MA, USA) maintained at 40°C. The flow rate was 0.25 mL/min with an injection volume of 2 μL. For LC-ESI (+)-MS analysis, the mobile phases were 0.1% formic acid in acetonitrile (C) and 0.1% formic acid in water (D), with the following gradient: 0–1 min, 2% C; 1–9 min, 2–50% C; 9–12 min, 50–98% C; 12–13.5 min, 98% C; 13.5–14 min, 98–2% C; 14–20 min, 2% C. For LC-ESI (−)-MS analysis, the mobile phases were acetonitrile (A) and 5 mM ammonium formate (B), with the gradient: 0–1 min, 2% A; 1–9 min, 2–50% A; 9–12 min, 50–98% A; 12–13.5 min, 98% A; 13.5–14 min, 98–2% A; 14–17 min, 2% A (Zelena et al., 2009).

Mass spectrometric detection of metabolites was performed on an Orbitrap Exploris 120 (Thermo Fisher Scientific, USA) with an ESI ion source. Simultaneous MS1 and MS/MS acquisition (Full MS-ddMS2 mode, data-dependent MS/MS) was employed with the following parameters: sheath gas pressure, 30 arb; aux gas flow, 10 arb; spray voltage, 3.50 kV (ESI+) and − 2.50 kV (ESI-); capillary temperature, 325°C; MS1 range, m/z 100–1,000; MS1 resolving power, 60,000 FWHM; number of data-dependent scans per cycle, 4; MS/MS resolving power, 15,000 FWHM; normalized collision energy, 30%; dynamic exclusion time, automatic (Want et al., 2013).

## 4 Results

### 4.1 XTS improves learning and memory abilities and ameliorates brain pathological changes in APP/PS1 mice

The Morris water maze experiment demonstrated that over the course of the 5-day spatial navigation trial (Figure 1B), the average latency time decreased across all groups of mice. Compared to the NC group, the M group showed a smaller reduction in latency, indicating impaired learning ability in the M group. In contrast, the Tre group exhibited a greater reduction in latency compared to the M group, demonstrating the positive effect of XTS on the learning ability of APP/PS1 mice. After the 5-day spatial navigation trial, the efficacy of XTS on APP/PS1 mice was evaluated on the 6th day through a spatial probe trial. In this trial, APP/PS1 mice in the M group crossed the platform fewer times (*p* < 0.0001) (Figure 1C) and spent less time (*p* < 0.0001) and distance (*p* < 0.001) in the platform zone (Figures 1D,E), indicating impaired spatial memory (Figures 1F,G,H). Compared to the M group, the Tre group crossed the platform more frequently (*p* < 0.0001) and spent a higher percentage of time (*p* < 0.0001) and distance (*p* < 0.05) in the platform zone, indicating that XTS improved the spatial memory of APP/PS1 mice. These results suggest that XTS intervention significantly enhanced learning ability and spatial memory in APP/PS1 mice. HE and Nissl staining were employed to observe the effects of XTS on the pathological changes in the brain tissues of APP/PS1 mice. The hippocampus is crucial for learning and memory and is particularly vulnerable to damage in the early stages of AD (Mu and Gage, 2011).

HE staining results (Figure 1I) showed that in the NC group, the hippocampal neuronal cells were well-organized, spherical, with intact cell membranes and nuclei, and no significant swelling or necrosis. In contrast, the M group exhibited irregular neuronal cell sizes, uneven structures, and reduced cell numbers. Following XTS intervention, the Tre group showed restoration in the morphology and number of neuronal cells, indicating that XTS effectively reversed neuronal cell necrosis. Similarly, Nissl staining also demonstrated the therapeutic effect of XTS. Nissl staining results (Figure 1J) showed that in the Con group, neuronal cells were normal in size and number, with regular morphology and tightly arranged, with abundant Nissl bodies in the cytoplasm. In the M group, neuronal cells exhibited vacuolar changes or shrinkage, a significant reduction in number, fewer Nissl bodies, lighter staining, loose arrangement, and nuclear dissolution. After XTS intervention, neuronal cells in the Tre group were more regularly arranged, with an increased number of Nissl bodies, reduced cell swelling or shrinkage, and increased intracellular Nissl bodies.

**Figure 2:**
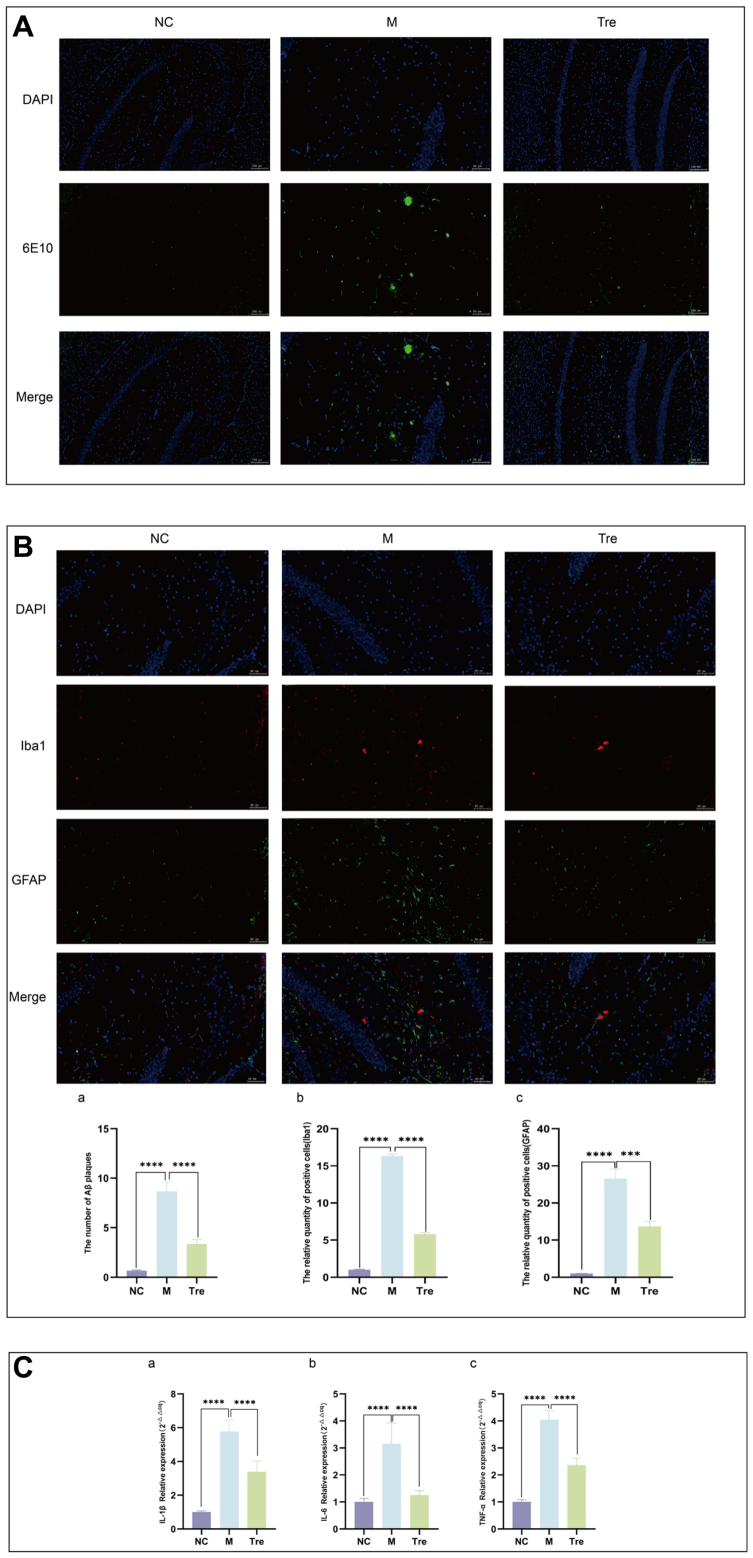
XTS reduced the pathological products in the brains of APP/PS1 mice. **(A)** Aβ immunofluorescence staining. **(B)** GFAP and Iba1 immunofluorescence staining. ^a^Quantification of Aβ plaques. ^b^Quantification of GFAP and Iba1 positive cells. **(C)** qPCR of inflammatory cytokines in mouse brain tissue (^a^IL-1β; ^b^IL-6; ^c^TNF-α). *p < 0.05; **p < 0.01; ***p < 0.001; ****p < 0.0001.

Immunofluorescence was used to detect Aβ accumulation in the brains of APP/PS1 mice using the 6E10 antibody (Figure 2A) and the activation of astrocytes and microglia using GFAP and Iba1 antibodies, respectively (Figure 2B). The results showed that compared to the NC group, the M group had increased Aβ deposition and greater activation of astrocytes and microglia, which were aggregated. Following XTS intervention, Aβ deposition and the activation of astrocytes and microglia in the brains of APP/PS1 mice were significantly reduced. These findings indicate that XTS can effectively mitigate pathological changes in the brains of APP/PS1 mice, reducing Aβ deposition and the activation of astrocytes and microglia. qPCR was used to measure the levels of inflammatory cytokines in the brain tissues of mice. The results (Figure 2C) showed that compared to the NC group, the M group had upregulated expression of IL-1β, IL-6, and TNF-α. Following XTS intervention, the expression levels of IL-1β, IL-6, and TNF-α were significantly reduced (*p* < 0.05). These results indicate that XTS lowers the levels of inflammatory cytokines in the brain tissues of APP/PS1 mice.

### 4.2 XTS alters the composition of gut microbiota in APP/PS1 mice

**Figure 3:**
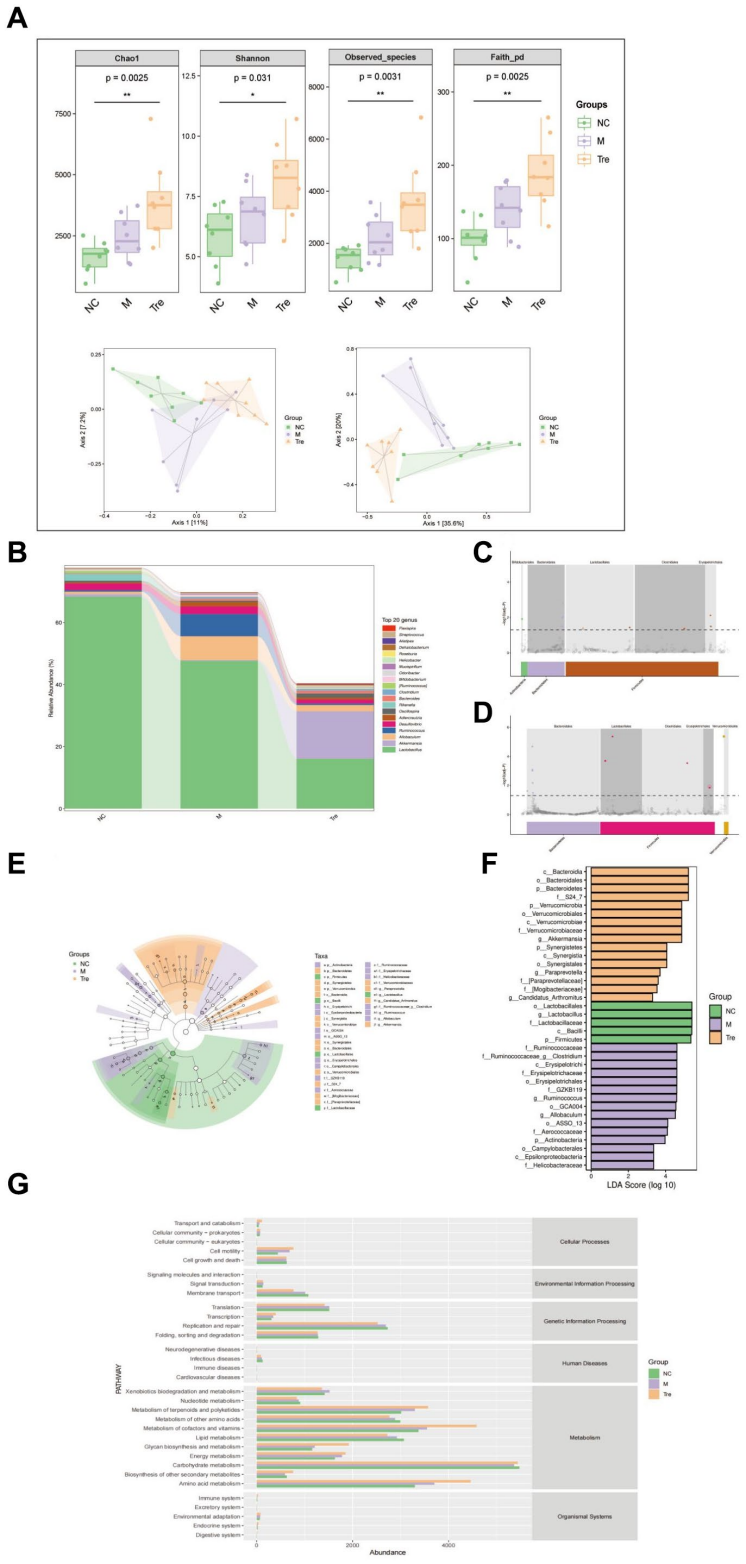
XTS improved the diversity and abundance of gut microbiota in APP/PS1 mice. **(A)** Alpha and beta diversity analysis of 16S rDNA sequencing. **(B)** Bar chart of species composition at the genus level. **(C)** Manhattan plot of different ASV/OTU based on metagenomeSeq (M vs. NC). **(D)** Manhattan plot of different ASV/OTU based on metagenomeSeq (Tre vs. M). **(E)** Display of intergroup differences in taxonomic units based on the classification tree. **(F)** Bar chart of LDA effect size of marker species. **(G)** Predicted KEGG secondary functional pathways.

To investigate whether XTS alters the gut microbiota composition in APP/PS1 mice and exerts anti-AD effects, we performed 16S rRNA gene sequencing on fecal samples from the NC, M, and Tre groups (eight samples per group). Alpha diversity indices were used to evaluate species richness and functional diversity among the groups (Figure 3A). The results indicated that compared to the M group, the Tre group showed higher Chao1 and observed species indices, suggesting that XTS increased the species richness and abundance of gut microbiota in APP/PS1 mice. The Shannon index was also significantly higher in the Tre group than in the M group, indicating an increase in gut microbiota diversity following XTS intervention. The Faith PD index, which measures phylogenetic diversity, was higher in the Tre group compared to the M group, suggesting improved systemic health and diversity of the gut microbiome in APP/PS1 mice after XTS treatment. Additionally, β-diversity analysis using PCoA revealed distinct microbial compositions among the three groups, indicating significant differences in microbial species.

To further assess the general composition of the gut microbiota in the three groups, we selected the top 20 most abundant genera and compared the taxonomic similarities. The results showed that Lactobacillus, Desulfovibrio, and Rikenella were the predominant genera in the NC group, while Lactobacillus, Allobaculum, and Ruminococcus were dominant in the M group, and Lactobacillus, Akkermansia, and Allobaculum were dominant in the Tre group (Figure 3B; Supplementary Table S1 in Supplementary material 1). Significant differential analysis revealed that, compared to the NC group, the M group exhibited significantly increased abundances of Bifidobacteriales, Bacteroidales, Lactobacillales, Clostridiales, and Erysipelotrichales (Figure 3C). In contrast, compared to the M group, the Tre group showed significant increases in the abundances of Bacteroidales, Lactobacillales, Clostridiales, Erysipelotrichales, and Verrucomicrobiales (Figure 3D). Kruskal-Wallis rank-sum tests identified significantly different species among the groups, and Wilcoxon rank-sum tests were used for pairwise comparisons. Linear discriminant analysis (LDA) was employed to classify the data and evaluate the contribution of significantly different species. The results (Figures 3E,F; Supplementary Table S2 in Supplementary material 1) indicated that the most contributory differential species varied among the groups: Ruminococcaceae in the M group, Bacteroidia in the Tre group, and Lactobacillales in the NC group.

In functional predictive analysis, PICRUSt2 was used to map the gut microbiota to potential functions. KEGG functional predictions based on 16S rRNA data were compared among the groups (Figure 3G; Supplementary Table S3 in Supplementary material 1). The results showed that carbohydrate metabolism and biosynthesis of other secondary metabolites were reduced in the M group compared to the NC group, but were restored in the Tre group. Xenobiotics biodegradation and metabolism were elevated in the M group compared to the NC group, but were downregulated in the Tre group. These findings suggest that XTS exerts its anti-AD effects by modulating the abundance and function of gut microbiota in APP/PS1 mice, enhancing carbohydrate metabolism and the biosynthesis of secondary metabolites, and downregulating xenobiotics biodegradation and metabolism.

### 4.3 XTS reverses metabolic disturbances in APP/PS1 mice

#### 4.3.1 XTS reverses metabolic disturbances in the gut of APP/PS1 mice

We further conducted LC–MS-based untargeted metabolomics on gut and brain tissues to investigate the effects of XTS on metabolites in APP/PS1 mice. The quality control of the LC–MS untargeted metabolomics was satisfactory (Supplementary material 2).

**Figure 4:**
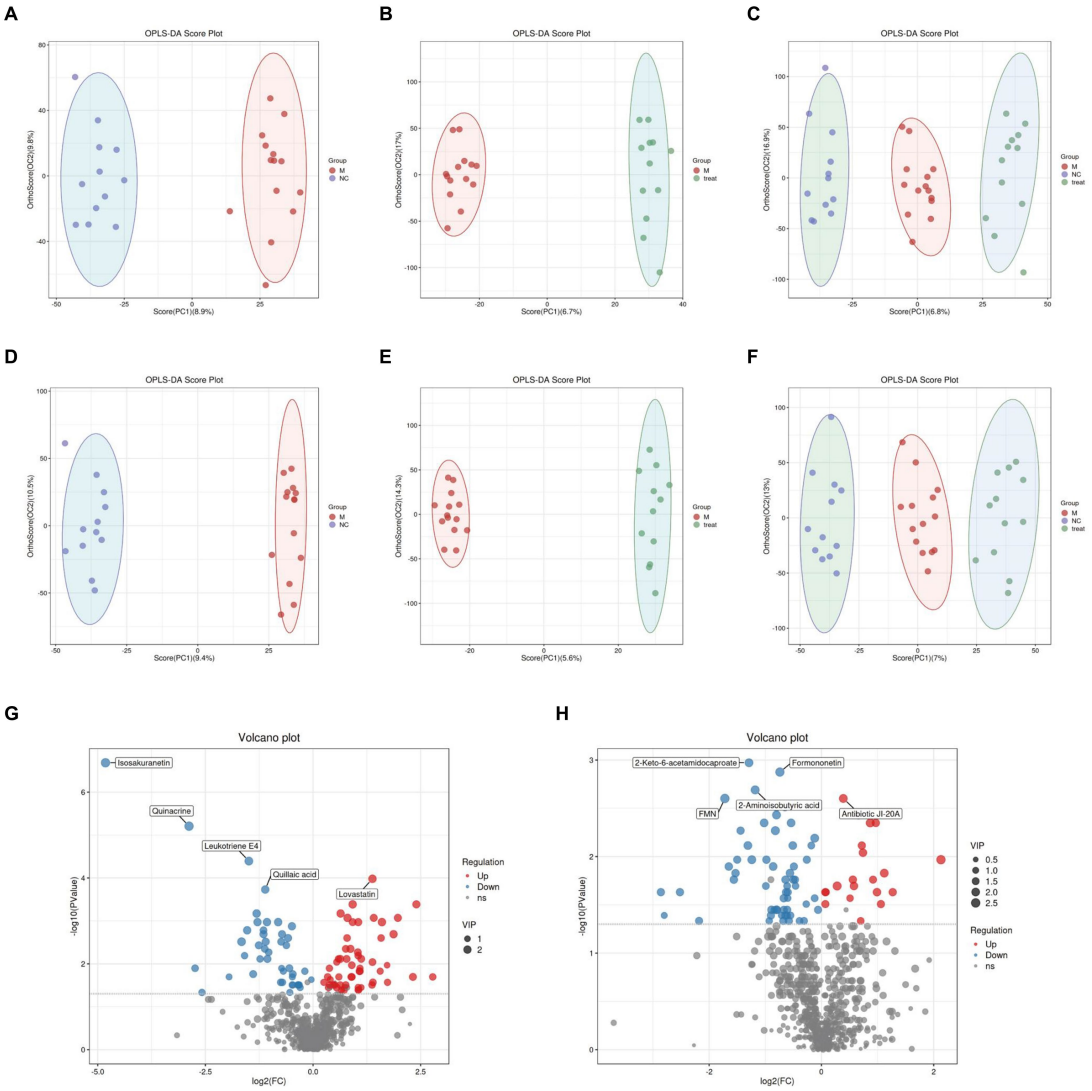
Gut metabolomics analysis-1. **(A)** OPLS-DA analysis of M group vs. NC group in positive ion mode. **(B)** OPLS-DA analysis of Tre group vs. M group in positive ion mode. **(C)** OPLS-DA analysis of NC, M, and Tre groups in positive ion mode. **(D)** OPLS-DA analysis of M group vs. NC group in negative ion mode. **(E)** OPLS-DA analysis of Tre group vs. M group in negative ion mode. **(F)** OPLS-DA analysis of NC, M, and Tre groups in negative ion mode. **(G)** Volcano plot of differential metabolites in gut tissue between M and NC groups. **(H)** Volcano plot of differential metabolites in gut tissue between Tre and M groups.

In the LC–MS untargeted gut tissue metabolomics, the detected metabolites were analyzed using OPLS-DA. OPLS-DA (Figures 4A–F) revealed distinct and differentially separated metabolite profiles among the three groups in both positive and negative ion modes, indicating significant metabolic disturbances in APP/PS1 mice that were partially restored by XTS treatment. Using Student’s t-test (*p* < 0.05), we visualized differential metabolites between groups with -log10 (*p* value) and log2 (Fold Change) values. We found 703 differential metabolites between the NC and M groups, with 100 metabolites showing significant differences (55 upregulated and 45 downregulated) (Figure 4G; Supplementary Table S1 in Supplementary material 3). Between the Tre and M groups, 703 metabolites were differentially expressed, with 78 showing significant differences (20 upregulated and 58 downregulated) (Figure 4H; Supplementary Table S2 in Supplementary material 3).

**Figure 5:**
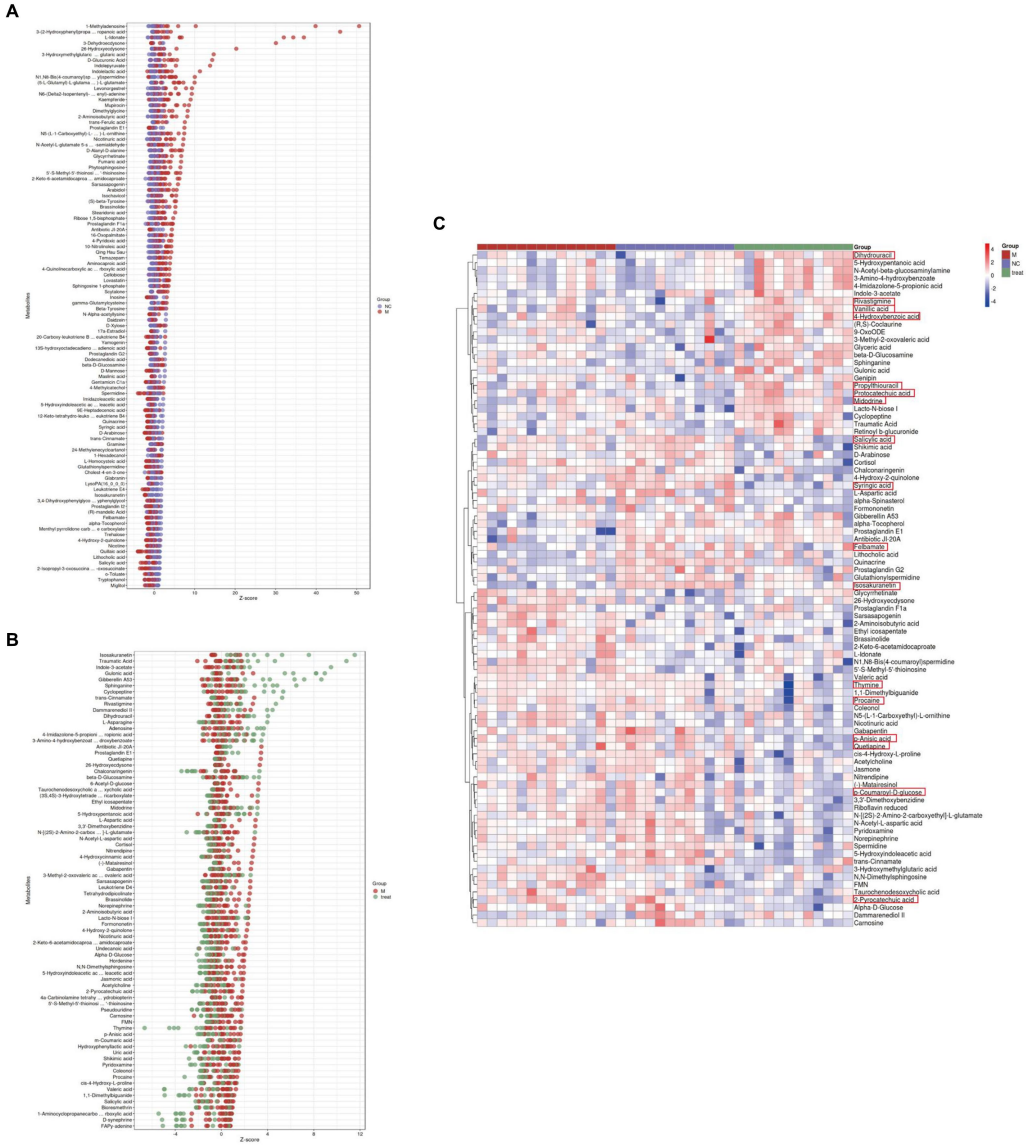
Gut metabolomics analysis-2. **(A)** Z-score plot of differential metabolites in brain tissue between M and NC groups. **(B)** Z-score plot of differential metabolites in brain tissue between Tre and M groups. **(C)** Heatmap of hierarchical clustering analysis among the three groups.

We calculated the ratios of the quantitative values of the differential metabolites and used Z-scores for differential analysis. The Z-scores, based on the relative content of metabolites, revealed that compared to the NC group, the M group exhibited significant differences in Quillaic acid (decreased) and 1-Methyladenosine (increased) (Figure 5A). Compared to the M group, the Tre group showed significant differences in Thymine (decreased) and Isosakuranetin (increased) (Figure 5B). Quillaic acid belongs to the category of Alcohols and Polyols within Carboxylic Acids and Derivatives, while 1-Methyladenosine is a Purine Nucleoside. Thymine is a Pyrimidine within Diazines, and Isosakuranetin is a 1-Benzopyran within Benzene and Substituted Derivatives. Cluster analysis revealed improvements in various differential metabolites in Diazines and Benzene and Substituted Derivatives following XTS intervention (Figure 5C; Supplementary Table S3 in Supplementary material 3). These findings suggest that XTS may exert anti-AD effects by modulating related metabolic processes of Diazines and Benzene and Substituted Derivatives.

#### 4.3.2 XTS reverses metabolic disturbances in the brain of APP/PS1 mice

**Figure 6:**
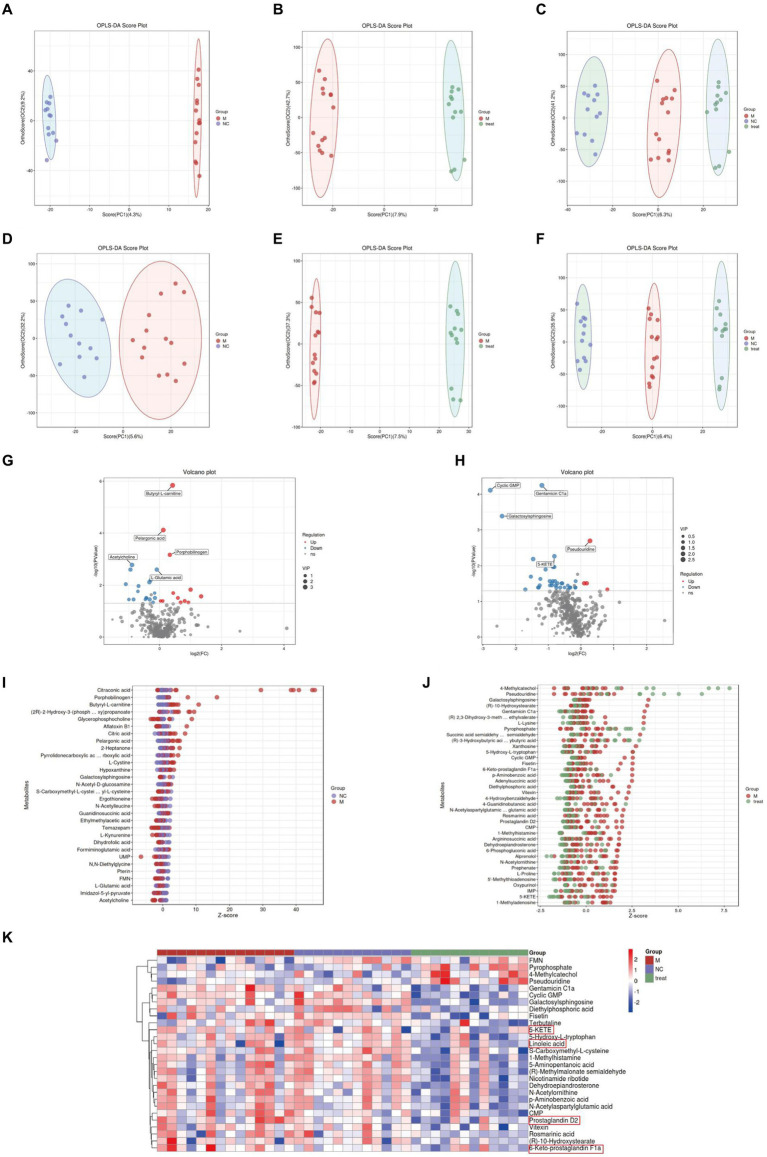
Brain metabolomics. **(A)** OPLS-DA analysis of M group vs. NC group in positive ion mode. **(B)** OPLS-DA analysis of Tre group vs. M group in positive ion mode. **(C)** OPLS-DA analysis of NC, M, and Tre groups in positive ion mode. **(D)** OPLS-DA analysis of M group vs. NC group in negative ion mode. **(E)** OPLS-DA analysis of Tre group vs. M group in negative ion mode. **(F)** OPLS-DA analysis of NC, M, and Tre groups in negative ion mode. **(G)** Volcano plot of differential metabolites in brain tissue between M and NC groups. **(H)** Volcano plot of differential metabolites in brain tissue between Tre and M groups. **(I)** Z-score plot of differential metabolites in brain tissue between M and NC groups. **(J)** Z-score plot of differential metabolites in brain tissue between Tre and M groups. **(K)** Heatmap of hierarchical clustering analysis among the three groups.

In the LC–MS untargeted brain tissue metabolomics, the detected metabolites were subjected to OPLS-DA. OPLS-DA (Figures 6A–F) showed clear and distinct separation of metabolites among the three groups in both positive and negative ion modes, indicating significant metabolic disturbances in APP/PS1 mice that were partially restored by XTS treatment. Using Student’s t-test (*p* < 0.05), we visualized the differential metabolites between groups with -log10 (*p* value) and log2 (Fold Change) values. We found 397 differential metabolites between the NC and M groups, with 30 showing significant differences (13 upregulated and 17 downregulated) (Figure 6G; Supplementary Table S4 in Supplementary material 3). Between the Tre and M groups, 397 metabolites were differentially expressed, with 38 showing significant differences (4 upregulated and 34 downregulated) (Figure 6H; Supplementary Table S5 in Supplementary material 3).

We calculated the ratios of the quantitative values of the differential metabolites and used Z-scores for differential analysis. The Z-scores revealed that compared to the NC group, the M group exhibited significant differences in Glycerophosphocholine (decreased) and Citraconic acid (increased) (Figure 6I). Compared to the M group, the Tre group showed significant differences in 5-KETE (decreased) and 4-methylcatechol (increased) (Figure 6J). Citraconic acid and 5-KETE belong to Fatty Acyls within Fatty Acids and Conjugates, which were elevated in the M group but reduced in the Tre group. These findings indicate that XTS intervention significantly affected the levels of Fatty Acyls in the brain tissues of APP/PS1 mice, with a notable reduction in 5-KETE following XTS treatment. Cluster analysis revealed improvements in various Fatty Acyls metabolites following XTS intervention (Figure 6K; Supplementary Table S6 in Supplementary material 3). These results suggest that XTS may exert anti-AD effects by modulating the metabolic processes related to Fatty Acyls.

### 4.4 XTS modulates metabolic pathways in APP/PS1 mice

#### 4.4.1 XTS modifies gut tissue metabolic pathways in APP/PS1 mice

**Figure 7:**
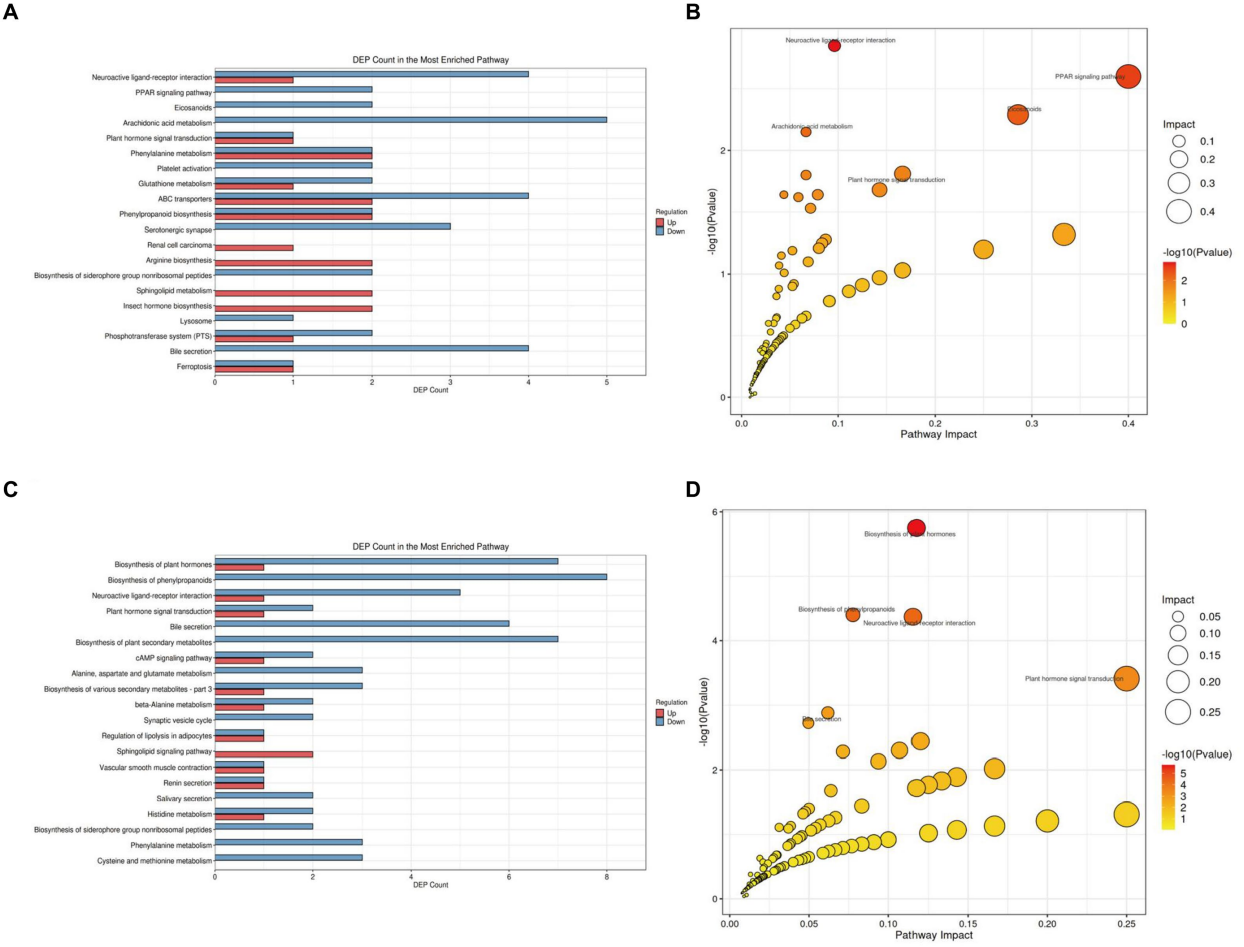
KEGG pathway analysis of gut tissue metabolites in APP/PS1 mice. **(A)** Statistical plot of differential molecules in KEGG pathways between M and NC groups. **(B)** Bubble chart of metabolic pathway analysis between M and NC groups. **(C)** Statistical plot of differential molecules in KEGG pathways between Tre and M groups. **(D)** Bubble chart of metabolic pathway analysis between Tre and M groups.

To further elucidate the functional roles of these differential metabolites and their impact on the host, we classified and annotated the identified differential metabolites using the KEGG database. This allowed us to determine their functional characteristics and identify major biochemical and signal transduction pathways. Compared with the NC group (Figure 7A; Supplementary Table S1 in Supplementary material 4), the M group exhibited downregulation of five metabolites within the Arachidonic acid metabolism pathway, which was the pathway with the most downregulated metabolites. Comprehensive analysis of the pathways involving differential metabolites (including enrichment and topology analysis) revealed key pathways with the highest correlation to metabolic differences (Figure 7B). The Neuroactive ligand-receptor interaction pathway emerged as the most correlated with metabolic differences, featuring differential metabolites such as Prostaglandin I2, 12-Keto-tetrahydro-leukotriene B4, Leukotriene E4, Sphingosine 1-phosphate, and L-Homocysteic acid.

Compared with the M group (Figure 7C; Supplementary Table S2 in Supplementary material 4), the Tre group showed significant alterations in the Biosynthesis of phenylpropanoids pathway, with eight differentially expressed metabolites, making it the pathway with the highest number of differential metabolites. Key metabolites in this pathway included L-Aspartic acid, Alpha-D-Glucose, trans-Cinnamate, Shikimic acid, Salicylic acid, Indole-3-acetate, 1-Aminocyclopropanecarboxylic acid, and Jasmonic acid (Figure 7D). The Neuroactive ligand-receptor interaction pathway also displayed significant metabolic differences between the Tre and M groups, with differential metabolites including L-Aspartic acid, Adenosine, Norepinephrine, Cortisol, Acetylcholine, and Leukotriene D4.

#### 4.4.2 XTS modifies brain tissue metabolic pathways in APP/PS1 mice

**Figure 8:**
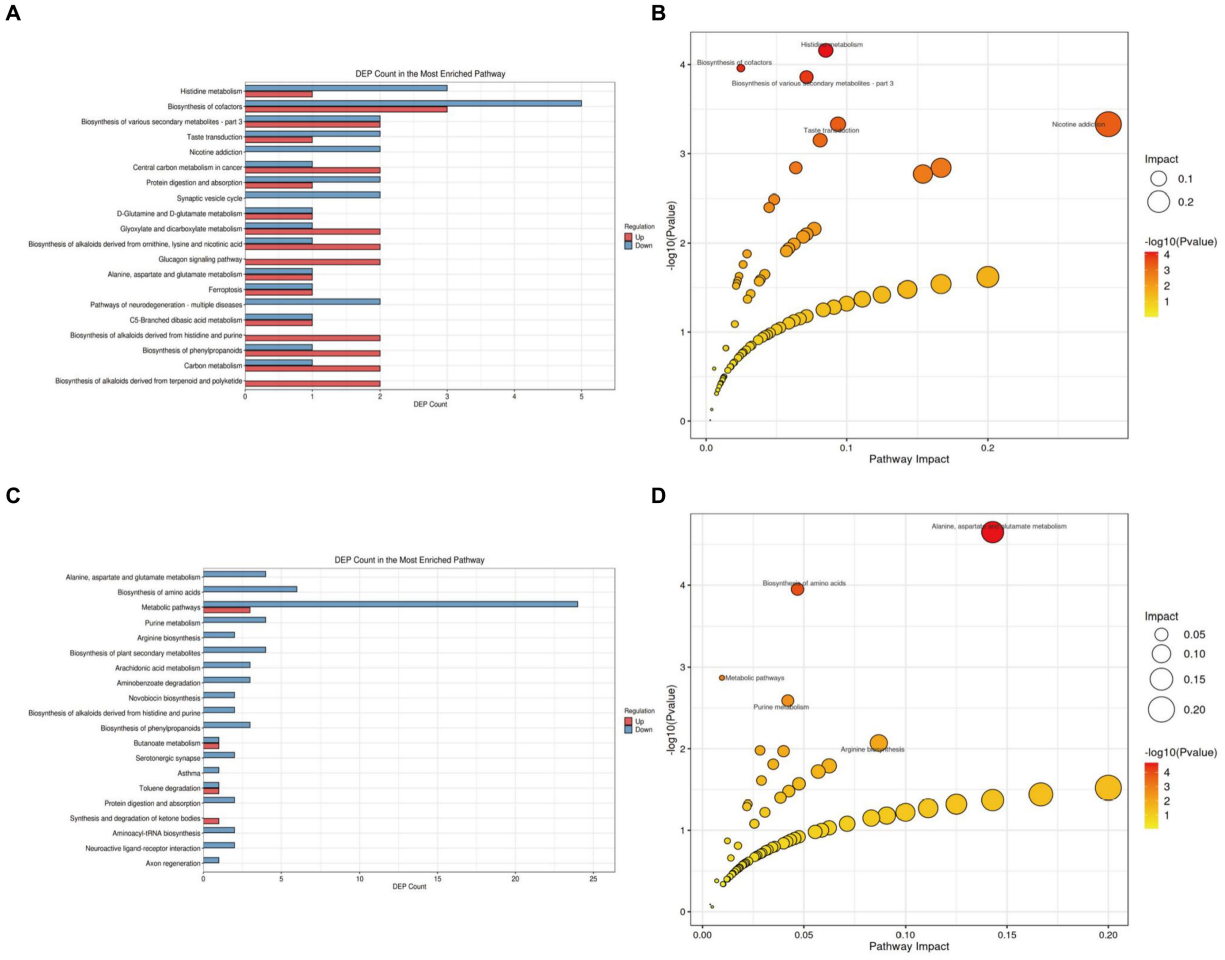
KEGG pathway analysis of brain tissue metabolites in APP/PS1 mice. **(A)** Statistical plot of differential molecules in KEGG pathways between M and NC groups. **(B)** Bubble chart of metabolic pathway analysis between M and NC groups. **(C)** Statistical plot of differential molecules in KEGG pathways between Tre and M groups. **(D)** Bubble chart of metabolic pathway analysis between Tre and M groups.

We further analyzed the alterations in metabolic pathways in the brain tissues of APP/PS1 mice. Compared with the NC group (Figure 8A; Supplementary Table S3 in Supplementary material 4), the M group showed downregulation of five metabolites and upregulation of three metabolites in the Biosynthesis of cofactors pathway, making it the pathway with the most differentially expressed metabolites. Enrichment and topology analyses identified Histidine metabolism as the key pathway most correlated with metabolic differences (Figure 8B), with differential metabolites including L-Glutamic acid, Formiminoglutamic acid, Imidazol-5-yl-pyruvate, and Ergothioneine.

Compared with the M group (Figure 8C; Supplementary Table S4 in Supplementary material 4), the Tre group exhibited downregulation of 24 metabolites and upregulation of three metabolites in the Metabolic pathways, the pathway with the most differentially expressed metabolites. Further analysis revealed that the Alanine, aspartate, and glutamate metabolism pathway had the highest correlation with metabolic differences, featuring key metabolites such as Succinic acid semialdehyde, Argininosuccinic acid, Adenylsuccinic acid, and N-Acetylaspartylglutamic acid (Figure 8D).

### 4.5 XTS improves the interactions between gut microbiota and brain tissue metabolites in APP/PS1 mice

**Figure 9:**
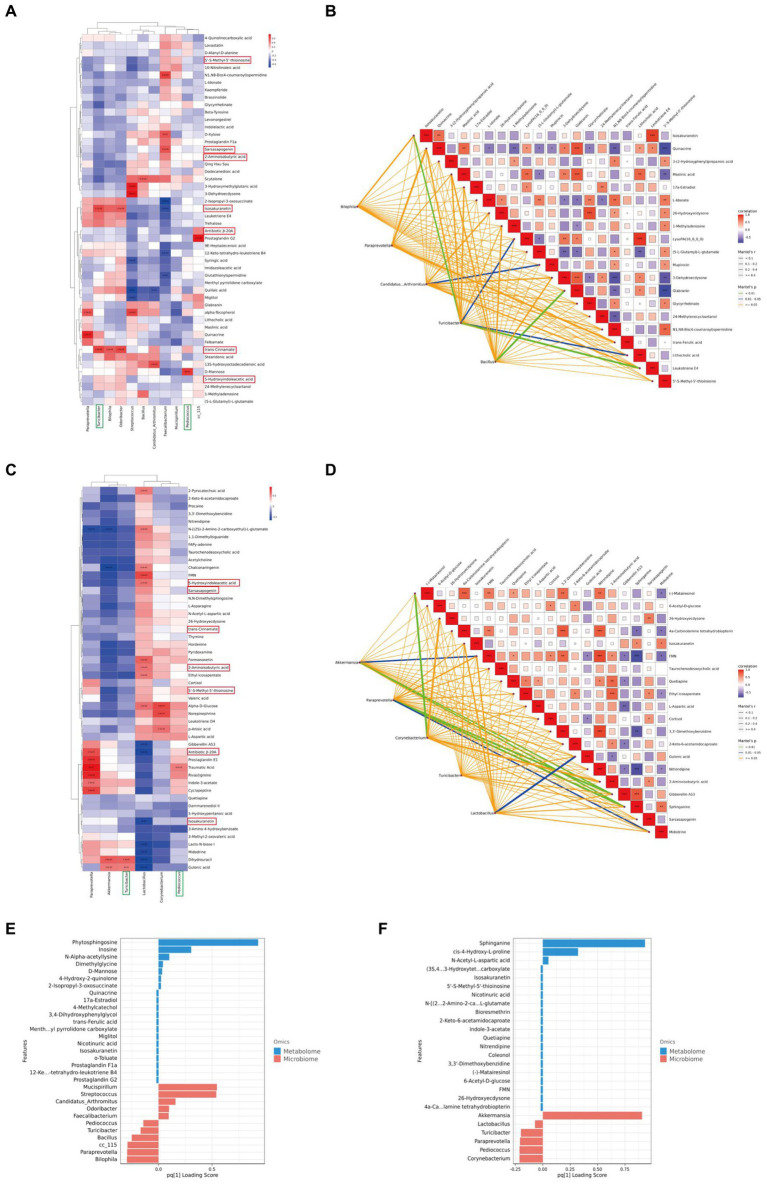
Joint analysis of metabolomics and 16S rDNA. **(A)** Spearman correlation analysis between NC and M groups. **(B)** Mantel Tests analysis between NC and M groups. **(C)** Spearman correlation analysis between M and Tre groups. **(D)** Mantel Tests analysis between M and Tre groups. **(E)** O2PLS analysis between NC and M groups. **(F)** O2PLS analysis between M and Tre groups.

To further investigate the impact of XTS on gut microbiota and metabolites and its potential anti-AD effects, we performed a Spearman correlation analysis between gut microbiota and brain tissue metabolites (Figures 9A,C; Supplementary material 5). The study identified seven common differential metabolites between the M group & NC group and the Tre group & M group (highlighted in red in Figure 9), which include 5’-S-Methyl-5′-thioinosine, Sarsasapogenin, 2-Aminoisobutyric acid, Isosakuranetin, Antibiotic JI-20A, trans-Cinnamate, and 5-Hydroxyindoleacetic acid. Additionally, two common gut microbes were identified (highlighted in green in Figure 9): Turicibacter and Pediococcus. In the comparison between the M group and the NC group, Turicibacter showed a positive correlation with two metabolites, Isosakuranetin and trans-Cinnamate, while Pediococcus was positively correlated with D-Mannose (*p* < 0.05). In the comparison between the Tre group and the M group, Turicibacter was positively correlated with Dihydrouracil and Gulonic acid, and Pediococcus with Traumatic Acid. In the Mantel tests (Figures 9B,D; Supplementary material 6), we demonstrated the correlation between the top 20 metabolites and the distance matrices of the top 5 microbes. In the M group, Turicibacter showed a strong positive correlation with four brain tissue metabolites: Leukotriene E4, Isosakuranetin, Lithocholic acid, and LysoPA(16_0_0_0). Following XTS intervention, in the Tre group, both Akkermansia and Paraprevotella displayed a strong positive correlation with two brain tissue metabolites: Sphinganine and Gibberellin A53. Subsequently, we employed Two-way Orthogonal Partial Least Squares (O2PLS) analysis to more precisely identify key regulatory phenomena. The O2PLS analysis (Figures 9E,F; Supplementary material 7) revealed that, in the comparison between the M group and the NC group, the brain tissue metabolite Phytosphingosine and the gut microbe Mucispirillum exhibited significant importance. In the comparison between the Tre group and the M group, the brain tissue metabolite Sphinganine and the gut microbe Akkermansia showed notable significance.

Among these findings, Akkermansia, which displayed significant importance post-XTS intervention, showed strong correlations with brain tissue metabolites, suggesting it as one of the most affected gut microbiota by XTS. Similarly, Sphinganine emerged as a highly significant metabolite, strongly correlated with various gut microbes, indicating it as one of the most influenced metabolites by XTS intervention.

## 5 Discussion

AD is a common neurodegenerative disorder among the elderly, with its prevalence rising due to global population aging (Breijyeh and Karaman, 2020). Current research suggests that physiological processes such as metabolism, endocrine function, and immunity can link gut microbiota to the brain via the MGBA, exerting regulatory effects (Yuan et al., 2022). XTS, derived from the dried stems of the traditional Chinese medicinal plant Xuetong, has shown anti-inflammatory and metabolic regulatory properties (Yu et al., 2022) and may exert anti-AD effects through the MGBA.

In this study, the Morris water maze test revealed that XTS can enhance learning and memory in APP/PS1 mice. Histological examinations using HE and Nissl staining indicated that XTS preserved neuronal morphology in the brains of APP/PS1 mice and prevented Nissl body dissolution. Immunofluorescence assays showed that XTS intervention significantly reduced Aβ plaque deposition and downregulated the activation of astrocytes and microglia in the brain tissue of APP/PS1 mice, suggesting that XTS decreases the production of pathological products and reduces neuroinflammation, as confirmed by qPCR detection of inflammatory markers.

16S rDNA sequencing demonstrated that XTS increased the abundance of gut microbiota in APP/PS1 mice, with significant differences in microbial species between the NC, M, and Tre groups, highlighting the substantial impact of XTS on gut microbiota. Notably, Akkermansia showed a significant increase in abundance in the Tre group, whereas it was scarcely present in the M group. Akkermansia is associated with anti-inflammatory effects and metabolic regulation (Dao et al., 2016), and it can modulate the MGBA by maintaining the intestinal mucosal barrier and regulating lipid metabolism (Dao et al., 2016; Gu et al., 2021). Studies have shown that Akkermansia can ameliorate diabetes-related AD and alleviate its pathological features (Qu et al., 2023). Similar increases in Akkermansia abundance have been observed in other studies involving drug interventions in AD mouse models (Li et al., 2023; Liu et al., 2023). Functional predictions based on the KEGG database from 16S rDNA data indicated that XTS intervention upregulated carbohydrate metabolism, which is notably disrupted in AD and can exacerbate neuroinflammation and neurotoxicity due to microglial dysfunction (Baik et al., 2019; Traxler et al., 2022). XTS demonstrated significant regulatory effects on this pathological phenomenon, suggesting it can enhance Akkermansia abundance in APP/PS1 mice, exert anti-AD effects through the MGBA, and significantly regulate aberrant carbohydrate metabolism.

In metabolomic studies of gut and brain tissues, we found that XTS intervention markedly increased the levels of Isosakuranetin in the gut. Isosakuranetin has notable anti-inflammatory properties and has shown efficacy in a mouse model of pneumonia induced by *Staphylococcus aureus* (Tian et al., 2023). Additionally, it reduces neurological damage in a rat model of cerebral infarction, demonstrating neuroprotective effects (Janyou et al., 2024), and exhibits anti-nociceptive properties in a rat model of peripheral neuropathy (Jia et al., 2017). In brain tissue metabolomic studies, Citraconic acid levels were elevated in APP/PS1 mice, while XTS intervention reduced 5-KETE levels. Both Citraconic acid and 5-KETE are fatty acyl metabolites involved in fatty acid metabolism, which is disrupted in AD (Huang and Mahley, 2014; Essayan-Perez and Südhof, 2023). 5-KETE acts as a chemotactic factor, inducing eosinophil migration and exacerbating neuroinflammation (Archambault et al., 2019), and it can regulate steroid hormone production and activate immune cells (Hughes et al., 2019). Post-XTS intervention, 4-methylcatechol levels increased in the brain, a compound with neuroprotective properties that stimulates the synthesis of brain-derived neurotrophic factor (BDNF) and nerve growth factor (NGF) (Fukuhara et al., 2012). 4-methylcatechol has demonstrated protective effects in models of neurological disorders, depression, pain, and recovery from motor and sensory nerve injury (Hsieh et al., 2009; Ishikawa et al., 2014; Gezginci-Oktayoglu et al., 2018). These findings suggest that XTS exerts anti-AD effects by upregulating Isosakuranetin in the gut, increasing 4-methylcatechol in the brain, and decreasing 5-KETE levels.

In combined analyses of gut microbiota and brain tissue metabolomics, Akkermansia again emerged as significant, showing a positive correlation with the metabolite Sphinganine. Sphinganine displayed notable importance in O2PLS analysis between the Tre and M groups. Studies have reported decreased urinary Sphinganine levels in AD patients (Li et al., 2013), and Sphinganine can regulate the balance between Th17 and Treg cells (Wang et al., 2023). KEGG database analysis indicated that the Neuroactive ligand-receptor interaction pathway showed high metabolic differentiation in gut tissues post-XTS intervention. Abnormalities in this pathway are closely associated with AD pathogenesis and play critical roles in regulating nervous system function and cellular communication (Pushparaj et al., 2022). In brain tissue, the Alanine, aspartate, and glutamate metabolism pathway was identified as the key pathway with the highest correlation with metabolic differences. D-Alanine positively correlates with behavioral symptoms in AD patients, while D-Glutamate and L-Glutamate negatively correlate (Lin et al., 2019; Ren et al., 2019). This pathway is essential for energy metabolism and neurotransmitter biosynthesis, supporting neuronal function (Wilkins and Trushina, 2018). N-Acetylaspartylglutamic acid (NAAG), a significant intermediate in this pathway, can convert to N-acetylaspartate (NAA) and ultimately aspartate, and is closely related to cognitive function and neuron survival (Schuff et al., 2006; Neale and Olszewski, 2019). AD patients show decreased levels of NAAG and NAA, correlating with cognitive impairment and neuronal loss (Jaarsma et al., 1994; Hollinger et al., 2019; Li et al., 2023). Additionally, this pathway is linked to neuroinflammation, with increased arginine levels observed in 3 × TgAD female mice, correlating with neuroinflammation (Liu et al., 2014). These findings suggest that XTS may link the gut and brain through the Neuroactive ligand-receptor interaction and Alanine, aspartate, and glutamate metabolism pathways, exerting anti-AD effects via the MGBA.

However, this study has limitations. The extraction process and yield of XTS require further research and improvement. The specific mechanisms and pathways of XTS’s anti-AD effects have not been fully elucidated, and the APP/PS1 double transgenic mouse model is only one type of AD rodent model, not encompassing all AD pathologies. Future studies should utilize more AD animal models, such as 5 × FAD and 3 × TgAD, to further explore the anti-AD effects of XTS.

## 6 Conclusion

Our study demonstrates that XTS effectively improves learning and memory function in APP/PS1 mice, ameliorates neuropathological changes and pathological products in brain tissue, and mitigates dysbiosis and metabolic disturbances in gut microbiota. XTS significantly modulates the abundance of Akkermansia microbiota and regulates the levels of metabolites such as Isosakuranetin, 5-KETE, 4-methylcatechol, and Sphinganine, exerting anti-AD effects via the MGBA.

## Data availability statement

The datasets presented in this study can be found in online repositories. The names of the repository/repositories and accession number(s) can be found at: https://www.ncbi.nlm.nih.gov/, PRJNA1117655.

## Ethics statement

The animal study was approved by the Ethics Committee of Hunan University of Chinese Medicine, LLBH-2021090804. The study was conducted in accordance with the local legislation and institutional requirements.

## Author contributions

ZS: Data curation, Resources, Writing – review & editing. JH: Data curation, Writing – original draft. WY: Investigation, Methodology, Supervision, Writing – review & editing. CH: Methodology, Supervision, Writing – review & editing. MY: Methodology, Software, Supervision, Writing – review & editing. PL: Supervision, Visualization, Writing – review & editing. ZL: Supervision, Writing – review & editing. GJ: Data curation, Writing – review & editing. SC: Funding acquisition, Resources, Writing – review & editing.

## Footnotes

^1^https://docs.qiime2.org/2019.4/tutorials/
