# Supplementary material for: Exploring the multifaceted therapeutic mechanism of Schisanlactone E (XTS) in APP/PS1 mouse model of Alzheimer’s disease through multi-omics analysis
Source: Front Microbiol. 2024 Jul 9;15:1440564. doi: 10.3389/fmicb.2024.1440564 (PMC11263214; doi:10.3389/fmicb.2024.1440564)
Supplement: Supplementary file 2 [file Table_2.DOCX]

**Quality Control in Untargeted Metabolomics Analysis Using LC-MS**

**Data inspection of samples was performed using base peak chromatograms (BPC).** The components eluted from chromatographic separation continuously entered the mass spectrometer, where the mass spectrum was scanned continuously to collect data. Each scan yielded a mass spectrum, and the ion with the highest intensity in each spectrum was selected and plotted continuously. The resulting plot used ion intensity as the vertical axis and time as the horizontal axis. The horizontal axis of the plot represents retention time, while the vertical axis represents ion intensity, with the upper right corner indicating the maximum ion intensity for each sample. Different colors represent different groups. The more similar the trends, the better the reproducibility, indicating reliable results (Figure 1-A, B).

**To obtain reliable and high-quality metabolomics data, quality control (QC) is necessaryFigure 1 Quality Control in LC-MS Untargeted Metabolomics Analysis.** (A. Base Peak Chromatogram (BPC) in positive ion mode. B. BPC in negative ion mode. C. PCA score plot of QC samples in positive ion mode. D. PCA score plot of QC samples in negative ion mode. E. RSD distribution in QA results for positive ion mode. F. RSD distribution in QA results for negative ion mode.)[^[[1]](#endnote-1)^]. In this experiment, QC samples were used for quality control during LC-MS analysis. Theoretically, QC samples are identical; however, systematic errors during sample extraction and analysis can cause variations among QC samples. Smaller variations indicate higher method stability and better data quality, which is reflected in the PCA analysis plot by the tight clustering of QC samples, indicating good data reproducibility and reliable results (Figure 1-C, D).

**To identify biomarkers, the relative standard deviation (RSD) of potential feature peaks in QC samples must not exceed 30%.** Feature peaks that do not meet this criterion should be deleted. Therefore, based on quality control, quality assurance (QA) is usually performed to remove poorly reproducible feature peaks in QC samples^[1]^. This step ensures a higher quality dataset, which is more conducive to biomarker detection. In QC samples, approximately 65% of feature peaks have an RSD of less than 30%, indicating good data quality[^[[2]](#endnote-2)^](Figure 1-E, F).


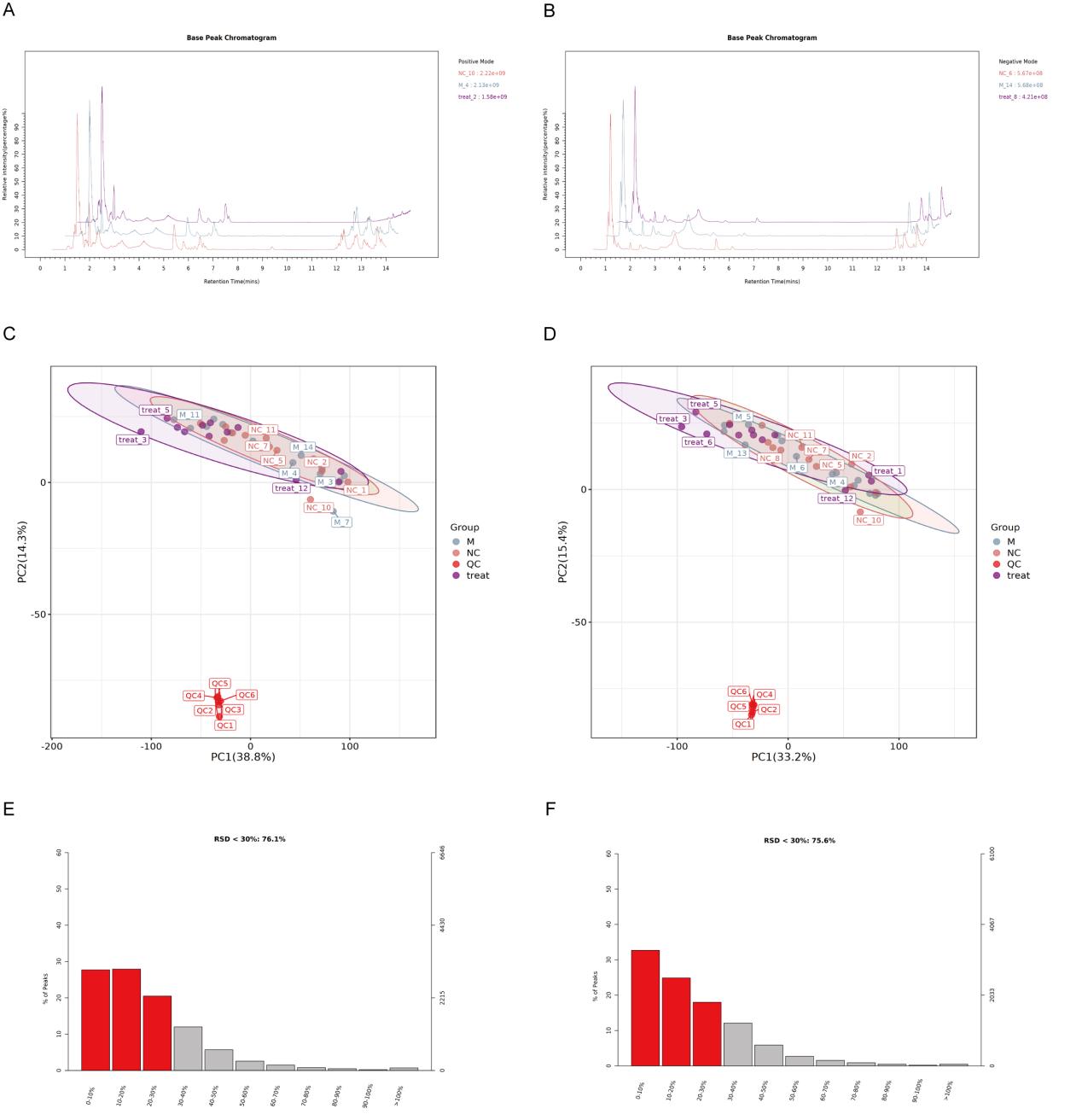


Figure 1 Quality Control in LC-MS Untargeted Metabolomics Analysis. (A. Base Peak Chromatogram (BPC) in positive ion mode. B. BPC in negative ion mode. C. PCA score plot of QC samples in positive ion mode. D. PCA score plot of QC samples in negative ion mode. E. RSD distribution in QA results for positive ion mode. F. RSD distribution in QA results for negative ion mode.)

1. [] Dunn W B, Broadhurst D, Begley P, et al. Procedures for large-scale metabolic profiling of serum and plasma using gas chromatography and liquid chromatography coupled to mass spectrometry.[J]. Nature protocols, 2011, 6(7):1060-83. [↑](#endnote-ref-1)
2. [] Want E J, Masson P, Michopoulos F, et al. Global metabolic profiling of animal and human tissues via UPLC-MS[J]. Nature Protocols, 2013, 8(1):17-32. [↑](#endnote-ref-2)
